# The synaptic hypothesis of schizophrenia version III: a master mechanism

**DOI:** 10.1038/s41380-023-02043-w

**Published:** 2023-04-11

**Authors:** Oliver D. Howes, Ellis Chika Onwordi

**Affiliations:** 1 Faculty of Medicine, Institute of Clinical Sciences (ICS), Imperial College London, London, W12 0NN UK; 2 Psychiatric Imaging Group, Medical Research Council, London Institute of Medical Sciences, Hammersmith Hospital, London, W12 0NN UK; 3 Department of Psychosis Studies, Institute of Psychiatry, Psychology & Neuroscience, King’s College London, London, SE5 8AF UK; 4 Centre for Psychiatry and Mental Health, Wolfson Institute of Population Health, Queen Mary University of London, London, E1 2AB UK

**Keywords:** Neuroscience, Physiology, Schizophrenia

## Abstract

The synaptic hypothesis of schizophrenia has been highly influential. However, new approaches mean there has been a step-change in the evidence available, and some tenets of earlier versions are not supported by recent findings. Here, we review normal synaptic development and evidence from structural and functional imaging and post-mortem studies that this is abnormal in people at risk and with schizophrenia. We then consider the mechanism that could underlie synaptic changes and update the hypothesis. Genome-wide association studies have identified a number of schizophrenia risk variants converging on pathways regulating synaptic elimination, formation and plasticity, including complement factors and microglial-mediated synaptic pruning. Induced pluripotent stem cell studies have demonstrated that patient-derived neurons show pre- and post-synaptic deficits, synaptic signalling alterations, and elevated, complement-dependent elimination of synaptic structures compared to control-derived lines. Preclinical data show that environmental risk factors linked to schizophrenia, such as stress and immune activation, can lead to synapse loss. Longitudinal MRI studies in patients, including in the prodrome, show divergent trajectories in grey matter volume and cortical thickness compared to controls, and PET imaging shows in vivo evidence for lower synaptic density in patients with schizophrenia. Based on this evidence, we propose version III of the synaptic hypothesis. This is a multi-hit model, whereby genetic and/or environmental risk factors render synapses vulnerable to excessive glia-mediated elimination triggered by stress during later neurodevelopment. We propose the loss of synapses disrupts pyramidal neuron function in the cortex to contribute to negative and cognitive symptoms and disinhibits projections to mesostriatal regions to contribute to dopamine overactivity and psychosis. It accounts for the typical onset of schizophrenia in adolescence/early adulthood, its major risk factors, and symptoms, and identifies potential synaptic, microglial and immune targets for treatment.

## Introduction

Schizophrenia is generally a severe mental illness with a lifetime prevalence of about 1% of the population worldwide [1], and a major cause of global disease burden [2]. Symptoms typically begin in late adolescence or early adulthood, and can be separated into three domains: (1) positive (e.g., hallucinations, delusions, paranoia and thought disorder), (2) negative (e.g., anhedonia, avolition, social withdrawal and thought poverty) and (3) cognitive (e.g., dysfunction in attention, working memory and executive function) [3]. The onset of the first psychotic episode is commonly preceded by a prodrome generally of 1–5 years and characterised by sub-clinical negative, cognitive and psychotic symptoms [4, 5].

In up to 20% of patients with schizophrenia, their illness shows a limited response to adequate trials of two different anti-psychotic drugs and clozapine [6] and treatments for negative symptoms and cognitive deficits remain an unmet clinical need [7]. Thus, there is a need to understand the pathoaetiology of schizophrenia to help identify new treatment targets.

**Fig. 1:**
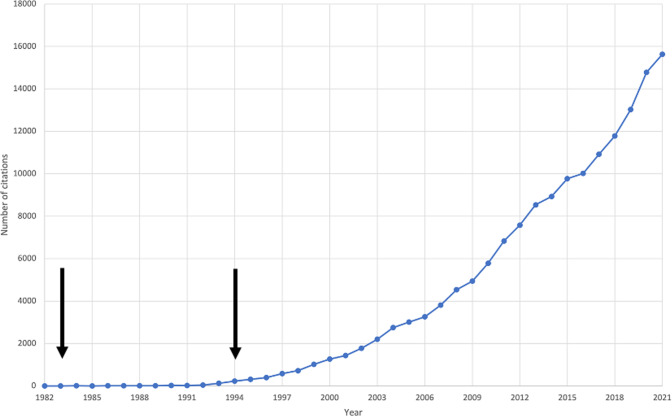
Citations per year, from 1982 to 2021, when searching for topic: (schizophrenia) and topic: (synap*) in Web of Science™. The first arrow indicates the year Feinberg’s hypothesis was published. The second arrow indicates the year Keshavan et al.’s hypothesis was published.

In 1982, Irwin Feinberg first proposed that a fault in synaptic elimination in adolescence is causal to schizophrenia [8]. The hypothesis was subsequently revised to propose that a combination of excessive pruning of cortical synapses in prefrontal circuits and insufficient pruning of subcortical synapses underlies the onset of schizophrenia [9]. Judged by the number of citations on the theme, the synaptic hypothesis of schizophrenia has stimulated substantial interest, particularly in the last few years (see Fig. 1).

However, since these iterations of the synaptic hypothesis of schizophrenia, new data from novel methods, such as induced pluripotent stem cell (iPSC) and genome-wide association studies (GWAS) and in vivo synaptic imaging, have emerged. Here, we update the synaptic hypothesis in light of this new evidence.

## The synaptic hypothesis version I

Feinberg proposed arguably the earliest version of the synaptic hypothesis of schizophrenia, stating that ‘too many, too few, or the wrong synapses are eliminated’ [8]. He speculated that this led to impaired neuronal integration, which resulted in auditory hallucinations, thought interference and the loss of self-boundaries observed in schizophrenia [8, 10].

Feinberg referred to ‘reduced synaptic density’ as an umbrella term for qualitative changes in synapses, such as reorganisation, as well as a quantitative reduction in the number of synapses. He cited four main lines of evidence in support of a role for synaptic alterations in schizophrenia. First, Feinberg observed that EEG wave amplitudes markedly increase in infancy, and decline substantially in adolescence, with little variation in adulthood. Second, brain metabolic rate, as measured by CMRO_2_ uptake, peaks in the first decade of life, declining rapidly through adolescence and early adulthood, before declining more slowly through the remaining adulthood [11]. Third, the degree of neuroanatomical plasticity observed in childhood, whereby the brain is capable of functional recovery from injury, is lost by adolescence. Fourth, Feinberg speculated that cognitive performance (termed ‘functional power’) peaks in adolescence. Feinberg observed that these electrophysiological, metabolic, anatomical and cognitive trajectories track trajectories for synaptic density, which peaks in childhood, before rapidly declining in late childhood and early adolescence [12]. He also observed that schizophrenia typically emerges in adolescence or early adulthood, thereby correlating temporally with the period of marked synaptic elimination. In addition, he noted that markers tracking synaptic trajectories (EEG wave amplitude and cognitive performance) are altered in schizophrenia.

Much of the evidence concerning synapses cited in version I of the hypothesis was indirect in nature, for instance, that cortical glucose metabolism is lower in schizophrenia than in controls. However, whilst lower glucose could reflect lower synaptic density, approximately 30% of cortical glucose metabolism supports non-signaling processes, unrelated to synaptic levels or activity [13]. This highlighted the need for more direct evidence of synaptic levels in schizophrenia.

## The synaptic hypothesis version II

In 1994, Keshavan et al. updated the synaptic hypothesis with new evidence for structural and metabolic abnormalities in schizophrenia, and revised it to propose excessive cortical pruning and insufficient subcortical pruning [9]. They also highlighted that there could be a failure to form synapses in the first place, excessive synaptic elimination later in neurodevelopment, excess synaptic production early in development, or a combination of these processes.

Keshavan et al. synthesised new evidence regarding normal neurodevelopment, by drawing on non-human primate and human data, which indicated a peak in cortical synaptic density in normal early postnatal development, followed by a sharp decline in synaptic density through puberty, and a slower decline in adulthood [14–17]. Keshavan et al. noted that these trajectories were consistent with Feinberg’s hypothesised synaptic trajectory in normal human neurodevelopment. However, they noted that the locus of synaptic elimination (both spatial, in relation to synapse type, laminar location, and regional variation, and temporal) had yet to be established.

Keshavan et al. built on the neurostructural foundations of Feinberg’s hypothesis, by incorporating new evidence from CT studies indicating that grey-white matter ratios reduce from childhood during normal development. They also incorporated new evidence for neurostructural alterations in schizophrenia, including post-mortem studies showing reduced brain volume, cortical thinning and sulcal enlargement [18, 19] and early MRI studies showing reduced frontal lobe volume [20, 21], greater frontal sulcal size [22], and reduced cortical grey matter volume [23, 24] in schizophrenia patients relative to healthy controls. The data were not all consistent, however, with other MRI studies failing to find significant differences in frontal or cerebral volume [25].

In addition, Feinberg speculated that synaptic elimination in adolescence may underlie reductions in cerebral metabolism and, when faulty, the onset of schizophrenia. This would suggest alterations in cerebral metabolism in schizophrenia. Keshavan et al.’s second version of the synaptic hypothesis of schizophrenia incorporated emerging evidence for frontal lobe hypometabolism, including positron emission tomography (PET), single-photon emission computed tomography, ^133^Xe inhalation, ^31^P-magnetic resonance spectroscopy, and cerebral blood flow studies indicating frontal hypometabolism [26–28], although again, these data were not unequivocal, with some studies failing to find evidence for hypofrontality [29].

Feinberg’s hypothesis lacked specificity in terms of the precise location of suspected synaptic alterations in schizophrenia. This was refined in the second synaptic hypothesis, which proposed excessive cortical pruning and insufficient subcortical pruning. The evidence for a failure of subcortical synaptic pruning was derived from individual MRI studies, which reported greater lenticular nucleus and left caudate volume in patients with schizophrenia [30, 31]. However, this could be an effect of anti-psychotic treatment and meta-analysis of these and subsequent studies have not found consistent evidence for subcortical alterations in anti-psychotic-free patients [32].

Critically, neither this nor version I proposed a mechanism for faulty synaptic elimination, or how this is linked to genetic or environmental risk factors. To address this, we review new lines of evidence and propose an updated version of the hypothesis.

## Evidence for synaptic changes in normal brain development

**Fig. 2:**
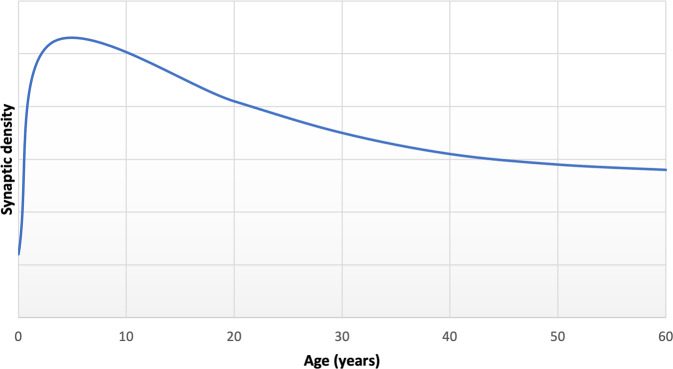
The normal trajectory of synaptic density over age, reflecting an initial phase of net synaptic production, followed by net synaptic elimination from late childhood/adolescence into early adulthood, and then relatively balanced synaptic elimination and production in middle age. Adapted from post-mortem data reported by Petanjek et al. for basal dendritic spine density of pyramidal cells from layer IIIc [34].

Multiple lines of evidence indicate that synapses undergo dramatic reorganisation through the course of life. Preclinical studies have found that normal development shows an early phase of net synaptic production followed by a phase of net synaptic elimination, and then comparatively balanced elimination and production leading to relatively stable synaptic levels in adulthood [15, 16, 33]. Consistent with this, human post-mortem studies show the greatest synapse density in early childhood, followed by intermediate levels during adolescence and early adulthood and lower, stable levels in adulthood (Fig. 2) [34]. Synaptic density reduces by approximately 40% from childhood through adolescence, with elimination particularly affecting glutamatergic synapses [9, 15, 16]. There was limited longitudinal in vivo imaging evidence for brain changes during human development when versions I and II of the hypothesis were proposed, but there have been a number of large studies since then. These show that cortical grey matter volumes increase and peak early in development [35], before undergoing a monotonic decrease into early adulthood [36–38]. Although beyond the scope of this review, it is important to recognise that findings from longitudinal imaging studies vary according to region, volumetric measure and developmental windows studied (see review [38]). The timing of these structural changes is broadly in line with the timing of synaptic changes seen in the preclinical and human post-mortem data on synaptic changes [36, 39], although this temporal association does not mean they are causally related (discussed further in the section on evidence for structural alterations in schizophrenia).

## The mechanisms governing synaptic elimination

**Fig. 3:**
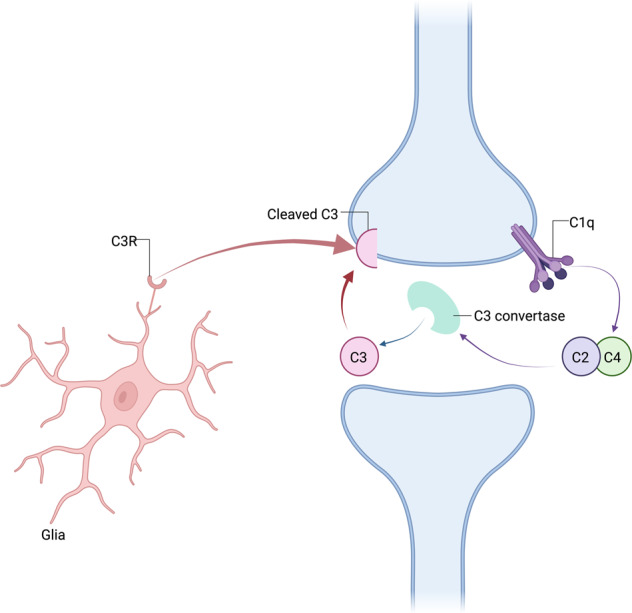
Mechanism for glia-mediated synaptic pruning via the classical complement signalling cascade. Complement component C1q interacting with binding partners triggers cleavage of the complement components C2 and C4, thereby promoting the generation of activated C3. Activated C3 induces synaptic phagocytosis by microglia. Other complement signalling pathways can also trigger phagocytosis.

Microglia are histiocytes that play a central role in synaptic elimination during normal brain maturation [40, 41]. During neurodevelopment, redundant synapses are tagged by complement proteins such as C1q (the cascade-initiating protein) and other complement proteins including C3 and C4, in a process which triggers the phagocytosis of the synapse by microglia (Fig. 3) [41, 42].

Adult mice overexpressing human C4 show increased microglial engulfment of synapses and reduced synapse density in the medial prefrontal cortex [43]. Similarly, C4-overexpressing mice show reduced spine number, and decreased spine turnover in juvenile mice, as well as abnormalities in glutamatergic cells [44]. In contrast, complement-dependent pruning is reduced by repeated firing of a synapse [42]. Thus, taken together, these lines of evidence show synaptic pruning is activity dependent and can be altered by genetic variants affecting the complement system.

In addition, astrocytes play both indirect and direct roles in synaptic elimination [45]. For example, C1q mRNA expression is dependent on factors secreted by astrocytes [42]. Moreover, astrocytes directly engulf synapses through the activation of phagocytic pathways necessary for normal neural circuit refinement [46].

Non-glial-related mechanisms also play a role in the elimination of synapses. For example, activation of the transcription factor myocyte enhancer factor 2 leads to ubiquitination of post-synaptic density protein 95 (PSD-95), which is then targeted for proteasomal degradation [47]. Stimulated synapses release molecules that change the actin skeleton in neighbouring dendritic spines, ultimately leading to their elimination [48]. Other molecules, such as semaphorin 7A, are released post-synaptically, and act in a retrograde manner to promote the elimination of pre-synaptic elements [49].

## Genetic and environmental risk factors for schizophrenia implicate synapses

There has been a massive expansion in the power of genetic association studies since earlier versions of the hypothesis. The latest genome wide association study (GWAS) includes 76,755 schizophrenia patients and 243,649 controls, and identified 287 common variant loci associated with schizophrenia. These associations implicate genes involved in synaptic organisation, differentiation and transmission, and post-synaptic terms, with additional enrichment of genes playing roles in synaptic transmission and signalling [50].

The most significant GWAS signal for schizophrenia in a sample predominantly of European origin lies in the major histocompatibility complex (MHC) and has been shown to link to complement expression, in particular to higher *C4A* brain expression levels [51, 52]. Complement-dependent pruning is reduced by repeated firing of a synapse and increased calcium signalling, as shown in the mouse visual cortex [42]. This suggests that the refinement of neural circuits via synapses could be altered by a genetic predisposition to impairments in the complement system and/or in glutamatergic signalling. However, it should be noted that a recent GWAS in people predominantly of East Asian ancestry did not find this association [53]. This highlights the need for more genetic studies in ethnically diverse groups to test the generalisability of findings [54]. Notwithstanding this, other risk factors for schizophrenia, such as stress, affect synaptic pruning (see summary in synaptic hypothesis version III section), and may account for aberrant synaptic pruning independent of genetic effects.

Brain co-expression network analyses coupled with gene ontogeny analyses have revealed that C4A expression levels are inversely associated with expression levels of synaptic genes, and that schizophrenia risk loci occur in synaptic pathways [52]. In addition, numerous other genetic loci associated with schizophrenia are linked to genes encoding proteins that mediate synaptogenesis [55], synaptic plasticity [56], spine formation and those involved in mechanisms of refining circuitry [55]. Animal models of some of these genetic risk factors have shown that they lead to lower synaptic marker levels [57–66].

Genetic loci linked to increased risk for schizophrenia are also involved in synaptic pathways during development. These include *VRK2*, encoding vaccinia-associated kinase 2, which is involved in neurodevelopmental microglia-mediated synaptic elimination [56], *CUL3*, encoding Culin-3, which is involved in neural development, glutamate receptor turnover and maintenance of excitation-inhibition balance [56, 67, 68], *KALRN*, encoding kalirin, which mediates dendritic spine formation [67, 68] and *CLSTN3*, encoding Calsyntenin-3, which promotes inhibitory and excitatory synaptic development [67, 68].

Early environmental insults, such as maternal infection, are risk factors for schizophrenia [69, 70], and animal models of antenatal infection or immune challenge show that these affect synaptic development, with some effects enduring into adulthood [71–73]. These seem to particularly affect synaptic development in glutamatergic neurons [72, 73].

Preclinical models of a number of other environmental risk factors for schizophrenia also show lower levels of synaptic markers. For example, studies of social isolation have identified that rats weaned on postnatal day 21 and subsequently reared in isolation for 8 weeks show lower medial prefrontal cortical and hippocampal dendritic spine density in adulthood (postnatal day 77) compared to controls reared in social groups [74]. Similarly, rats aged 28–32 days subsequently reared in individual caged environments for 30 days showed lower dorsolateral striatal dendritic spine density in adulthood compared to controls reared in complex environments containing social groups and objects [75]. A model of chronic social defeat stress has found that mice aged 8–10 weeks introduced to and attacked by unfamiliar resident mice show lower levels of prefrontal cortical pyramidal neuron apical dendritic spine density 30 days following the stress procedure compared to controls [76]. Numerous studies have identified that rodents subjected to chronic stress show changes in dendritic arborisation, with effects varying according to the brain regions studied [77–80]. Furthermore, studies of maternal immune activation models, which expose pregnant rodents to lipopolysaccharide [81] or polyribocytidylic acid (poly I:C) [82] on gestational day 9.5, have found lower levels of both pre-synaptic and post-synaptic markers in cortical brain regions as adults. This includes lower levels of pre-synaptic proteins such as synaptophysin, syntaxin-1 and synaptobrevin, and lower levels of post-synaptic proteins, including PSD-95 and SH3 and multiple ankyrin repeat domains proteins 1, 2 and 3, at postnatal days 52–54 [81], and also lower dendritic spine density at postnatal day 80 [82] compared to controls. These models show that risk factors acting at various developmental stages can lead to loss of synaptic markers. Similarly, lifetime stress has been linked to lower levels of dendritic spine density post-mortem in human cortical tissue [83]. It should be recognised that a range of brain and behavioural alterations are seen in these preclinical models, and it remains to be established that the synaptic changes are primary rather than secondary to other alterations. Furthermore, the effects of stress on dendritic spine density may show specificity in terms of brain region, spine type and timing of stressful events [76]. Nevertheless, taken together with the genetic studies, these findings indicate that a range of risk factors for schizophrenia affect synaptic levels to potentially increase vulnerability to the disorder.

The link between complement expression and brain structure and function has begun to be investigated. A recent imaging genetics study of a mixed sample of healthy control subjects (*n* = 46), patients with psychosis (*n* = 40) and individuals at clinical high risk for psychosis (*n* = 43) showed that levels of genotype-predicted brain *C4A* expression were positively associated with brain levels of translocator protein (TSPO, a marker expressed by glia), and negatively associated with hippocampal surface area [84]. However, there was no significant effect of clinical group on these relationships, indicating it is most likely a common mechanism. Further work in a large UK Biobank sample (n > 27,000) which excluded individuals with diagnoses of neurological or mental disorders identified that predicted *C4A* expression levels are negatively associated with cortical thickness in a number of brain regions implicated in schizophrenia pathogenesis, including the parahippocampal, insula, entorhinal, medial orbitofrontal and parts of the cingulate cortices [85]. Other work has investigated the relationship between complement markers and phosphorous magnetic resonance spectroscopy, which enables the quantification of membrane phospholipid precursors and catabolites [86] that are considered as proxies for the degree of neuropil contraction [87]. Higher *C4A* gene copy number has been shown to be directly associated with higher levels of catabolites in the inferior frontal cortex, and lower levels of precursors in the inferior parietal lobule, suggestive of greater neuropil contraction in these regions, in patients with schizophrenia [87]. In addition, higher C4A expression levels have been negatively associated with middle temporal cortex activation in healthy controls during an functional MRI (fMRI) visual processing task, and with episodic memory performance in healthy controls and patients with schizophrenia [88]. The findings discussed in this section thus show associations between diminished cortical volumes and thickness and altered brain activation and complement levels, consistent with a model that higher complement levels could underlie brain structural and functional changes in schizophrenia, although, importantly, it remains to be established if complement leads to the changes. It should also be recognised that there are inconsistencies in the relationship between *C4A* and brain imaging measures [85, 87]. Thus, further work is needed to investigate if complement expression underlies brain structural and functional changes, and to test if there is a link between complement expression and in vivo synaptic marker levels in patients with schizophrenia.

## Post-mortem synaptic markers in schizophrenia

Since earlier iterations of the synaptic hypothesis of schizophrenia, a wealth of post-mortem evidence for lower levels of markers of synaptic density in schizophrenia has accumulated.

Post-mortem studies have reported lower levels of a number of pre-synaptic markers in schizophrenia relative to controls, with moderate-to-large effect size lower levels of synaptophysin in the frontal cortex, cingulate cortex and hippocampus on meta-analysis [89]. Furthermore, a meta-analysis of post-mortem studies found lower levels of post-synaptic elements (comprising dendritic spine density, post-synaptic density and post-synaptic density (PSD) protein expression levels) in people with schizophrenia, with a moderate effect size in cortical tissues [90]. Subgroup analysis demonstrated that levels of post-synaptic elements were lower in cortical but not subcortical tissues [90]. Thus, findings of lower pre-synaptic and post-synaptic markers in separate meta-analyses of post-mortem studies are highly suggestive of cortical synaptic alterations in schizophrenia. These findings are supported by electron microscopy studies, which provide the gold standard means for directly measuring synaptic density, showing lower levels of axospinous [91] and axodendritic [92] synaptic density in the anterior cingulate cortex, perforated (principally glutamatergic) synapses in the striatum [93], and lower total synaptic density in the substantia nigra, particularly affecting symmetric (inhibitory) synapses [94] in tissue from patients with schizophrenia compared to controls. However, there are inconsistencies in the findings, potentially relating to differences in methodological approaches and the specific markers used [95]. Post-mortem studies in schizophrenia are also subject to a number of potentially confounding factors, such as differences in lifetime anti-psychotic exposure, differences in cause of death and post-mortem interval [95]. Moreover, post-mortem studies are highly labour intensive, therefore limiting the number of subjects and regions investigated in the individual studies. These issues limit the generalisability of findings. Critically, they cannot provide conclusive evidence of synaptic density changes in the living brain, or when they occur in the illness.

## Findings from neuronal cultures derived using induced pluripotent stem cells (iPSC) from patients

A significant technological advance since the earlier versions of the synaptic hypothesis has been the ability to use stem cells from patients to derive neuronal cultures. This enables neuronal development to be studied in brain tissue with the same genetic background as patients [96]. As seen in Table 1, studies implementing these methods show evidence for both pre- and post-synaptic deficits, such as lower synaptic vesicle 2 (SV2) and synapsin I puncta density and synaptic vesicle release, and lower levels of post-synaptic markers including PSD-95 protein levels and dendritic spine density. They also show functional alterations in synaptic signalling in neurons derived from people with schizophrenia compared to controls (Table 1).

**Table 1:** Studies of synaptic markers in iPSC-derived neuronal cultures from schizophrenia patients relative to healthy controls.

Author	Year	*n* (HC/SCZ)	SCZ genetic/phenotypic variant	Source	Generated cell types	Synaptic marker	Significant differences in synaptic markers seen in schizophrenia relative to control line	Reference
Brennand et al.	2011	4/4	1 with childhood-onset schizophrenia	Fibroblasts	Neurons	Neuronal connectivity	↓	[177]
						Neurite number	↓	
						PSD-95 protein levels	↓	
						Glutamate receptor expression	↓	
Wen et al.	2014	3/1	Frameshift mutation of DISC1	Skin fibroblasts	Forebrain MAP2AB+ neurons	SV2+ puncta density	↓	[178]
						Synaptic vesicle release	↓	
Shao et al.	2019	14/14	Idiopathic	Fibroblasts	Cortical interneurons	Inhibitory synapse density on interneurons	↓	[179]
						Excitatory synapse density on interneurons	↔	
Sellgren et al.	2019	4/4 (generating neurons); 18/13 generating microglia	Idiopathic	Monocytes	Microglia and neurons	Synapse elimination in neural cultures and synaptosomes	↑	[97]
						Dendritic spine density in neural lines co-cultured with microglial lines	↓	
Kathuria et al.	2019	9/9	Idiopathic	Fibroblasts	Cortical interneurons; co-cultured with excitatory cortical pyramidal neurons	NLGN2	↓	[180]
						Gephyrin levels	↓	
						Synaptic puncta density	↓	
Grunwald et al.	2019	3/3	Idiopathic	Skin fibroblasts	Neurons	PSD-95 spot density	↓	[181]
						Colocalised PSD-95 and vGlut	↔	
						Neurite length	↓	
						Number of synapsin I puncta on glutamatergic neurons	↓	
						Number of Homer I puncta on glutamatergic neurons	↓	
						Number of synapsin I puncta on GABAergic neurons	↓	
						Number of gephyrin puncta on GABAergic neurons	↓	
						Number of colocalised pre-synaptic and post-synaptic markers	↓	

*PSD-95* post-synaptic density protein 95, *SV2* synaptic vesicle glycoprotein 2, *DISC1* disrupted in schizophrenia protein 1, *MAP2AB1* microtubule-associated protein 2, *PV* parvalbumin, *VGAT* vesicular GABA transporter, *Vglut1* vesicular glutamate transporter 1, *NLGN2* neuroligin-2, *RELN* reelin gene.

**Fig. 4:**
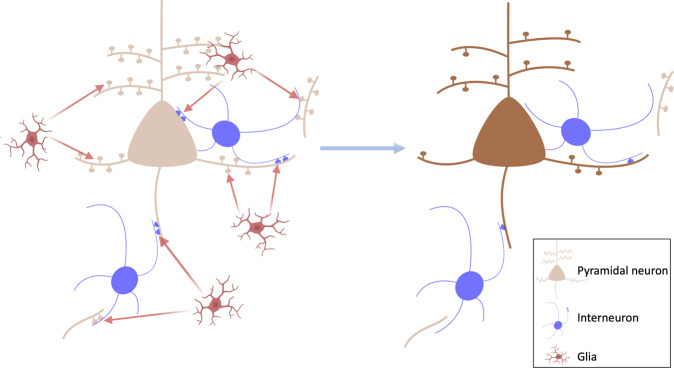
Showing the mechanism potentially leading to aberrant synaptic pruning in schizophrenia. Left: potential model of glia-mediated elimination of synapses in schizophrenia. This could affect glutamatergic synapses, including dendritic spines and collaterals that synapse onto inhibitory interneurons, as well as inhibitory synapses onto pyramidal neurons. Right: loss of synapses on pyramidal neurons and inhibitory interneurons, which could disrupt pyramidal neuron function and lead to negative and cognitive symptoms.

Importantly, recent iPSC models investigating synapse-glia interactions in vitro have demonstrated elevated, complement-dependent elimination of synaptic structures [97], highlighting a potential mechanism for excessive synaptic pruning by glia in schizophrenia [98, 99], summarised in Fig. 4. Thus, the evidence from patient-derived neural cultures indicates a failure to form and/or preserve synapses in early neurodevelopment in schizophrenia. Whilst beyond the scope of our review, it should be noted that in addition to synaptic alterations, iPSC models also show evidence for alterations in other aspects of neuronal development [100]. The role of these in the synaptic alterations remains to be determined.

It is also important to appreciate the limitations of evidence obtained through patient-derived neuronal lines. As these neural cells are cultured in vitro, they do not adequately reflect the impact of environmental factors on neurobiology in schizophrenia [101]. Furthermore, the limited maturity of derived neural cells, methodological variability in processes for cellular reprogramming and brain cell generation, and genetic background variations may affect findings [96]. These considerations highlight the need for in vivo studies with other techniques in patients.

## Evidence for altered brain structure in schizophrenia

There has been a step-change in the in vivo imaging evidence for altered brain structure in schizophrenia since versions I and II of the synaptic hypothesis were elaborated with, now, well over a hundred studies across brain regions and phases of illness [32, 102]. Meta-analyses of findings in patients show well-replicated evidence for lower cortical grey matter volumes (Hedges *g* ~0.26–0.66 [32], Cohen’s *d* ~0.31–1.09 [102]), and lower or unaltered subcortical volumes (Hedges *g* = ~0.11–0.46 [32], Cohen’s *d* = 0.18 [102]) relative to healthy controls across illness phases from the first episode. Moreover, there is now substantial evidence for similar patterns of lower grey matter volumes and cortical thinning in people at clinical high risk for schizophrenia, and that this is particularly marked in those who go on to develop the disorder [103, 104]. Meta-analysis also indicates that cortical thickness in fronto-temporal brain regions is lower in schizophrenia from the onset of the disorder (*z* ~1.94–3.25) and in clinical high-risk subjects (*z* ~1.01) [105].

In addition, studies have compared age-related brain structure in patients with controls. Cross-sectional imaging studies suggest that there is an accelerated age-related decline of grey matter volume in schizophrenia patients compared with controls [106]. This has been directly tested in longitudinal imaging studies, with meta-analyses of these indicating greater grey matter loss over time in patients compared to controls [107, 108]. Furthermore, cross-sectional imaging has identified that these deficits start early in disorder [106], and longitudinal studies have demonstrated that elevated rates of cortical grey matter loss are associated with conversion from clinical high risk to a psychotic illness [104, 109]. Moreover, there is an inverse relationship between cerebral volume and symptom severity in schizophrenia [110–112]. These observations implicate neurostructural alteration in the development of schizophrenia, raising the question as to the cellular and molecular basis of these changes.

A key question is, thus, whether structural MR imaging changes could be due to synaptic loss. Kassem et al. addressed this key question by combining structural MRI and confocal microscopy [113]. They observed grey matter volume loss on MRI in the anterior cingulate cortex and hippocampus of stressed mice, and reduced dendritic volume and spine density, in the absence of changes in the number or volumes of neuronal soma, astrocytes or oligodendrocytes. Moreover, there was a strong linear relationship (*R*^2^ > 0.9) between dendritic volume loss and MRI-estimated grey matter volume loss [113]. Similarly, Keifer et al. deployed voxel-based morphometry and confocal microscopy to investigate the effects of an auditory fear conditioning paradigm in mice. They found increased grey matter voxel intensity in the conditioned mice relative to the controls in the auditory cortex, amygdala and insula; concurrent increases in dendritic spine density in the auditory cortex; a positive relationship between dendritic spine density and grey matter voxel intensity; no change in neuronal nuclei density; and no relationship between nuclei density and grey matter voxel intensity [114]. These data show that synaptic changes can lead to changes in grey matter volumes measured by MRI, and indicate that stress can contribute to this. It should be noted, however, that Keifer et al. did not identify a significant change in auditory cortical thickness, suggesting dendritic spine density is less strongly linked to cortical thickness than to grey matter density.

Whilst cortical neuronal number remains largely unchanged in schizophrenia [115], post-mortem evidence shows lower cortical neuropil [116], cortical dendritic spine density [90, 117–120], spine plasticity markers and synaptic vesicle protein levels relative to controls (as discussed above; also see [89, 121–124]). Thus, taken together, this preclinical and post-mortem evidence indicates that synaptic changes could contribute to the neurostructural alterations seen in schizophrenia, although it does not prove it.

There are limitations to interpreting structural MRI findings as indicative of synaptic alterations in schizophrenia. Neuronal and glial number and size, and vasculature, as well as synaptic elements, may contribute to the grey matter signal ([114, 125], and as discussed in [126]). Factors such as anti-psychotic treatment and movement artefacts could also confound case–control differences [126, 127]. Thus, alterations in non-synaptic factors may contribute to or even account for structural MRI findings in schizophrenia, and so there has been a pressing need to develop in vivo imaging measures that are specific to synapses. This is reviewed in the next section.

## In vivo evidence for lower synaptic markers in schizophrenia

A major limitation of the synaptic hypothesis was the lack of evidence for synaptic alterations in patients with schizophrenia in vivo. However, investigating synaptic density in the living human brain has recently been made possible by the development of PET radioligands, such as [^11^C]UCB-J, that are specific for synaptic vesicle glycoprotein 2A (SV2A) [128]. SV2A is ubiquitously expressed in pre-synaptic terminals, and is a marker of synaptic terminal density [128]. Non-human primate studies demonstrate a strong positive relationship between [^11^C]UCB-J volumes of distribution and in vitro SV2A levels measured using western blots (*r* > 0.8) and binding assays (*r* > 0.9) [128]. Displacement studies using levetiracetam [128, 129], a drug which binds selectively to SV2A [130], show the [^11^C]UCB-J signal is largely blocked, indicating high specificity of [^11^C]UCB-J to SV2A. This evidence indicates that [^11^C]UCB-J is a specific marker of SV2A levels. SV2A, one of three isoforms of SV2, is expressed throughout the brain and is present in GABAergic and glutamatergic pre-synaptic nerve terminals [131]. Furthermore, SV2A levels are strongly, positively correlated with synaptophysin levels in the brain (*r* > 0.95) [128], which is reduced in disorders associated with synaptic loss, and is widely used as a marker of synaptic density [132]. Moreover, SV2 shows lower variability in terms of copy number per synaptic vesicle than synaptophysin [133]. [^11^C]UCB-J PET has demonstrated sensitivity to synaptic reductions in temporal lobe epilepsy and Alzheimer’s disease [128, 134], showing it is able to detect alterations in disorders in which loss of synaptic density is expected.

The first [^11^C]UCB-J PET study in patients with schizophrenia found that [^11^C]UCB-J volume of distribution was lower in patients compared to healthy volunteers in the frontal and anterior cingulate cortices with large effect sizes, and possibly lower in the hippocampus as well [135]. This study also found evidence for lower synaptic density in subcortical regions in patients with schizophrenia, in contrast to the prediction from version II of the synaptic hypothesis. These findings have since been independently replicated [136]. To our knowledge, SV2A levels have not been studied post-mortem in schizophrenia in the frontal or anterior cingulate cortices or hippocampus, although a post-mortem study found lower SV2A transcript levels in the cerebellar cortex in schizophrenia compared to controls [137]. Both PET studies were in patients with chronic illnesses who were taking anti-psychotic drugs. However, neither study found a relationship between anti-psychotic exposure and [^11^C]UCB-J binding, and a rodent study showed anti-psychotic drug exposure had no effect on SV2A protein or SV2A radioligand binding levels, indicating anti-psychotic treatment is unlikely to explain lower SV2A levels in schizophrenia [135].

In healthy volunteers, [^11^C]UCB-J binding and glutamate levels are directly associated in the anterior cingulate cortex and hippocampus, consistent with the high proportion of glutamatergic synapses there [138]. However, no significant relationship is seen between [^11^C]UCB-J and glutamate measures in schizophrenia, suggesting a loss of glutamatergic synapses and/or a lower ratio of glutamatergic to GABAergic synapses in the disorder [138].

There are a number of considerations in interpreting the [^11^C]UCB-J signal as a marker of synaptic density. As a marker of SV2A levels, changes in [^11^C]UCB-J binding could reflect altered SV2A levels, and/or synaptic vesicle numbers and/or pre-synaptic terminal density and/or synaptic density. However, as discussed earlier, there is post-mortem evidence showing lower levels of a number of pre- and post-synaptic elements in schizophrenia. This includes lower levels of synaptophysin and other synaptic vesicle proteins [89, 121–124], lower transcript levels of SV2A [137], lower cortical dendritic spine density and other post-synaptic elements, [90, 117, 118, 120] and lower spine plasticity [139], in the context of unaltered neuronal numbers in schizophrenia [115]. When this post-mortem evidence is taken with the [^11^C]UCB-J findings, the most parsimonious explanation is, thus, that lower [^11^C]UCB-J binding reflects lower synaptic density in schizophrenia.

## Version III of the synaptic hypothesis of schizophrenia: a master mechanism

The evidence from the post-mortem and PET studies discussed above provides direct evidence for lower synaptic levels, particularly in frontal regions, in schizophrenia, whilst the iPSC studies show lower synaptic marker levels, synaptic signalling deficits and elevated microglial-mediated synaptic pruning in neurons derived from patients relative to controls. On top of this, the MRI imaging data in schizophrenia show a greater loss of grey matter than seen in normal neurodevelopment and functional dysconnectivity across brain regions, both starting early in the course of the disorder. A number of brain changes could account for these structural and functional imaging alterations. However, given the preclinical data indicating that synaptic loss can account for at least a proportion of grey matter volume reductions, and taken with the PET, iPSC and post-mortem findings, synaptic loss likely contributes to these structural and functional alterations.

**Fig. 5:**
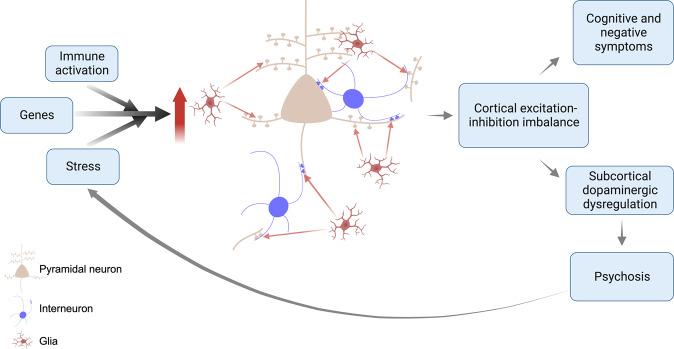
The synaptic hypothesis of schizophrenia version III. This is a multi-hit model in which genetic variants increase the vulnerability of synapses to elimination, and subsequent environmental risk factors such as stress, then induce aberrant glial-mediated pruning. Aberrant synaptic pruning leads to cortical excitation-inhibition imbalance, resulting in cognitive impairment and negative symptoms, and dysregulated projections to the striatum and midbrain. This leads to dopaminergic neuron disinhibition, and impairments in predictive learning and processing of sensory stimuli, causing psychotic symptoms. The stress of psychosis feeds back on this system to lead to further aberrant pruning.

Based on these lines of evidence, we propose a revised synaptic hypothesis, summarised in Fig. 5. The GWAS data link schizophrenia to risk variants involved in synapse formation (see earlier discussion), and a variant complement protein associated with increased microglial-mediated synaptic pruning, whilst the iPSC data indicate genetic risk translates into elevated microglial-mediated pruning and aberrant signalling in neurons derived from patients with schizophrenia. Schizophrenia is also associated with a number of environmental risk factors that are immune activators, such as maternal infections and obstetric complications [140], which have been shown to activate microglia so that they show an enhanced response to subsequent activation by later stressors in a process termed priming [141, 142]. These environmental and genetic risk factors may, thus, increase the vulnerability of the individual to an excessive response to subsequent microglial activation. Exposure to psychosocial stressors, such as physical or emotional abuse, bullying or other adverse life events, also increases the risk of schizophrenia [3]. Animal studies show that stressors that recapitulate aspects of these risk factors, such as repeated exposure to dominant animals, activate microglia and lead to synaptic pruning [76, 143–146]. Thus, genetic vulnerability translates into the aberrant formation of synapses and higher levels of complement proteins that tag synapses for elimination by glia, whilst environmental risk factors for schizophrenia may prime glia early in development and reactivate them later to lead to aberrant pruning of synapses by glia. Thus, we propose a multi-hit model, with both early and late risk factors converging to lead to synapse dysfunction and aberrant glial-mediated synaptic pruning. As microglia show priming, exposure to early risk factors may affect the timing of illness onset by enhancing their response to subsequent risk factors, such as psychosocial stress. Priming could thus account for findings that people exposed to early developmental environmental risk factors, such as obstetric complications, may be at increased risk of early onset of schizophrenia [140] because primed glia show an enhanced response to subsequent activation by stress.

A key question is how aberrant synaptic function and pruning could contribute to cognitive and negative symptoms. Schizophrenia is associated with lower grey matter in cortical regions, such as the frontal cortex, that play key roles in goal-directed behaviours, working memory and other processes that underlie these symptoms [32]. A balance between excitation and inhibition is critical to ensure the optimal signal-to-noise ratio in cortical neuronal arrays [147]. We propose that aberrant synaptic pruning leads to excitation-inhibition imbalance in cortical arrays and a lower signal-to-noise ratio [148]. This would impair function in cortical regions, contributing to the cognitive impairments and negative symptoms seen in schizophrenia. It could also account for neurofunctional alterations, such as findings of frontal hypometabolism [149], or fMRI studies finding altered cortical function and connectivity in schizophrenia [150–156]. However, it is important to note that numerous factors contribute to fMRI measures of functional connectivity, including blood flow, blood volume and cerebral metabolic rate [157], and the extent to which synaptic factors contribute to fMRI connectivity measures and cerebral glucose metabolism remains undetermined. Moreover, it remains to be established whether aberrant synaptic pruning affects particular excitatory or inhibitory synapses.

Another central question is how aberrant synaptic function and pruning could contribute to psychotic symptoms. Multiple lines of evidence indicate that overactivity in mesostriatal dopamine neurons underlies the development of psychotic symptoms by dysregulating their role in predictive learning and the assignment of confidence to the detection of sensory stimuli [158]. Preclinical evidence indicates that disrupting cortical excitation-inhibition balance, for example, using ketamine, can lead to mesostriatal dopamine overactivity [159–161] and result in elevated striatal dopamine synthesis capacity, as seen in patients with schizophrenia [161, 162]. Moreover, activating inhibitory interneurons in cortical brain regions may prevent hyperdopaminergia in a sub-chronic ketamine rodent model [161]. Thus, we propose that cortical excitation-inhibition imbalance due to aberrant synaptic function and/or pruning in turn may contribute to the dysregulation of neurons that project to the striatum and midbrain to disinhibit dopaminergic neurons in these regions, impairing predictive learning and processing of sensory stimuli to lead psychotic symptoms (for review see [163]). It should also be recognised that psychosis is, itself, intensely stressful, which may feedback on the system to cause further glial-mediated synaptic pruning and, in turn, worsen symptoms; setting up a vicious cycle (Fig. 5). Supporting this potential effect of psychosis, there is some evidence that greater duration of untreated psychosis is associated with larger grey matter reductions, albeit this does not directly show synaptic changes and further work is needed to test this association further [164, 165].

This model could explain why schizophrenia is rare in childhood: during this period, the net production of synapses provides a buffer against overactive pruning and synaptic dysfunction. However, the normal developmental switch to net synaptic elimination in adolescence/early adulthood makes the system much more vulnerable to overactive pruning, unmasking the vulnerability to schizophrenia, and explaining its peak onset during this period.

Version III builds on the earlier versions, and we acknowledge a great debt to the many contributors to these. It extends them by incorporating new evidence to propose a mechanism that links risk factors to synaptic changes and then to symptoms. The new hypothesis is also a multi-hit model whereby genetics increase the vulnerability of synapses to elimination, and environmental risk factors act later on this vulnerable system to cause aberrant synapse-microglia interactions, resulting in synaptic dysfunction and excess microglia-mediated pruning, contributing to symptoms in disorder. This could be considered a master mechanism, as multiple risk factors may converge to lead to synaptic dysfunction and aberrant synaptic elimination which, in turn, has the potential to underlie many other pathophysiological findings in schizophrenia, including structural and functional brain imaging findings, and dopaminergic dysfunction. The hypothesis is falsifiable by, for example, showing that synaptic alterations are not associated with the worsening of symptoms.

The evidence outlined above has implications for developing new treatments. As synaptic elimination is a dynamic process governed by the complement system and glial activity, these could be novel treatment targets to restore normal synaptic organisation by, for example, reducing the activation of microglia.

## Gaps in the evidence and future directions

A key evidence gap is whether there are synaptic alterations at the first episode, or earlier in the development of schizophrenia. PET studies in clinical high risk and unmedicated, first-episode schizophrenia would be invaluable in testing this. The finding of an altered relationship between an SV2A marker and glutamate measures in patients requires replication and the development of more specific imaging probes of glutamatergic synapses to enable it to be directly tested. An evidential gap is thus the link between synaptic loss and excitation-inhibition imbalance, and whether the synaptic loss particularly affects specific synapses, such as glutamatergic synapses. This could be tested using multi-modal imaging in patients and in studies of neurons derived from patients. Furthermore, developing organoid models would permit the investigation of neurodevelopmental stages later than those captured by 2D iPSC models.

We have drawn on evidence from preclinical and clinical studies using a variety of markers related to synapses, including pre-synaptic elements, post-synaptic structures and dendritic spine densities, and considered these as indicative of synaptic levels. However, whilst changes in these could be consistent with altered synaptic levels, we cannot exclude that some could be altered in the absence of synaptic changes. This highlights the value of future studies including measures of both pre- and post-synaptic elements to confirm that differences reflect synaptic loss. A related consideration is that some lines of evidence, such as post-mortem findings of lower dendritic spine density in schizophrenia relative to controls, could be due to a failure to form synapses instead of, or in addition to, aberrant synaptic pruning. Longitudinal studies would help disambiguate these possibilities.

To our knowledge, there have not been post-mortem studies of SV2A protein levels in schizophrenia, and the study of SV2A transcript levels was in the cerebellar cortex so it remains unclear if there are SV2A protein alterations post-mortem in the regions where changes are seen in vivo [137]. Conducting post-mortem studies of SV2A would be useful to corroborate the PET findings, and to investigate the relationship between levels of SV2A and those of other pre-, as well as post-synaptic proteins. Moreover, synaptic loss alone is highly unlikely to account for all the grey matter volume changes in schizophrenia and changes in related structures, such as dendritic spine density, likely contribute as well (see [126] for discussion). Another gap in knowledge is, thus, the degree to which synaptic loss accounts for the structural and functional imaging findings in schizophrenia. Multi-modal imaging studies combining synaptic marker measures, such as [^11^C]UCB-J, and other modalities would be invaluable in assessing this.

The hypothesis proposes that glia are activated by environmental risk factors, such as stress. There is evidence for higher levels of markers associated with an activated glial phenotype in schizophrenia relative to controls from post-mortem studies (effect size ~0.7 on meta-analysis) [166], and that greater PET signal for TSPO, a protein expressed by activated glia, is associated with greater predicted complement levels in patients [84]. However, there are inconsistencies in the TSPO PET findings in schizophrenia (reviewed in [167]). Whilst the inconsistencies could be due to methodological issues [167, 168], inconsistencies could also be due to the timing of the scans in relation to glial activation. Supporting the latter, a mouse study found social defeat increased TSPO PET signal in cortical regions in a time-specific manner [169]. Studies using TSPO PET imaging and synaptic markers in preclinical models of schizophrenia risk factors would be useful to further test these links, whilst the development of new tools to image glia is needed to provide greater sensitivity and specificity [170].

We have highlighted a potential role of C4, and this is now supported by recent evidence that cerebrospinal fluid concentrations of C4A are elevated in two separate cohorts of people with schizophrenia, and inversely related to levels of a synaptic marker [171]. However, it is important to recognise that this is only one potential pathway and other immune pathways or alternative mechanisms may also be involved. Indeed, there may be several pathways to the abnormal synaptic formation and/or pruning and the relative importance of C4 in this is unknown. These issues need further investigation. It should also be recognised that the GWAS studies are predominantly in European samples, and a recent GWAS in a population from East Asia did not find an association between schizophrenia and the major histocompatability locus linked to C4 variants [53]. There is thus a need for studies to include more diverse populations to test generalisability. Moreover, schizophrenia shows heterogeneity in its clinical and other features [32, 172, 173], and may show pathophysiologically based sub-types associated with distinct clinical phenotypes, such as late onset, treatment resistance and substance dependence, and variable illness trajectories, including some that show recovery after one episode [174–176]. It is unlikely that anyone neurobiological hypothesis can account for all sub-types of schizophrenia. For this reason, it is a potential master mechanism, but may not be the only one. Thus, it is important to determine if the synaptic loss is seen in sub-types of schizophrenia as well as typical presentations. By the same token, studies comparing synaptic markers in patients with neurodevelopmental disorders that share some genetic and other risk factors, such as schizophrenia and bipolar disorder, would be useful to determine if they share a common underlying mechanism. Finally, the proposed relationship between synaptic alterations and striatal dopamine dysregulation remains to be tested in vivo. This aspect of the hypothesis could be falsified by showing there is no association between cortical synaptic indices and striatal dopaminergic measures.

## Conclusion

A considerable body of new evidence for synaptic loss in patients has grown over the four decades since Feinberg first proposed the synaptic hypothesis of schizophrenia. Crucially, there is now in vivo evidence for lower synaptic terminal levels in patients, a mechanism mediated by microglia that accounts for genetic and environmental risk factors for the disorder, and new understanding on how synaptic loss could contribute to symptoms. We have revised the hypothesis to account for these new data. We call it version III, as we anticipate elements may need revision as further evidence accumulates. Notwithstanding this, it has the potential to explain a number of key aspects of the epidemiology and clinical expression of schizophrenia, including the peak age of onset and symptoms, and identifies mechanisms and new potential targets for treatment.

### Disclaimer

The views expressed are those of the authors and not necessarily those of the National Health Services, the National Institute of Health Research, or the Department of Health and Social Care of the United Kingdom.

## References

[1] Saha S, Chant D, Welham J, McGrath J (2005). A systematic review of the prevalence of schizophrenia. PLoS Med.

[2] McCutcheon RA, Reis Marques T, Howes OD (2020). Schizophrenia – an overview. JAMA Psychiatry.

[3] Howes OD, Murray RM (2014). Schizophrenia: an integrated sociodevelopmental-cognitive model. Lancet.

[4] Cannon TD, Cadenhead K, Cornblatt B, Woods SW, Addington J, Walker E (2008). Prediction of psychosis in youth at high clinical risk: a multisite longitudinal study in North America. Arch Gen Psychiatry.

[5] Yung AR, McGorry PD (1996). The prodromal phase of first-episode psychosis: past and current conceptualizations. Schizophr Bull.

[6] Siskind D, Siskind V, Kisely S (2017). Clozapine response rates among people with treatment-resistant schizophrenia: data from a systematic review and meta-analysis. Can J Psychiatry.

[7] Kaar SJ, Natesan S, McCutcheon R, Howes OD (2020). Antipsychotics: mechanisms underlying clinical response and side-effects and novel treatment approaches based on pathophysiology. Neuropharmacology.

[8] Feinberg I (1982). Schizophrenia: caused by a fault in programmed synaptic elimination during adolescence?. J Psychiatr Res.

[9] Keshavan MS, Anderson S, Pettergrew JW (1994). Is schizophrenia due to excessive synaptic pruning in the prefrontal cortex? The Feinberg hypothesis revisited. J Psychiatr Res.

[10] Feinberg I (1978). Efference copy and corollary discharge: implications for thinking and its disorders. Schizophr Bull.

[11] Kety SS (1956). Human cerebral blood flow and oxygen consumption as related to aging. J Chronic Dis.

[12] Huttenlocher PR (1979). Synaptic density in human frontal cortex – developmental changes and effects of aging. Brain Res.

[13] Yu Y, Herman P, Rothman DL, Agarwal D, Hyder F (2018). Evaluating the gray and white matter energy budgets of human brain function. J Cereb Blood Flow Metab.

[14] Rakic P, Bourgeois JP, Eckenhoff MF, Zecevic N, Goldman-Rakic PS (1986). Concurrent overproduction of synapses in diverse regions of the primate cerebral cortex. Science.

[15] Bourgeois JP, Rakic P (1993). Changes of synaptic density in the primary visual cortex of the macaque monkey from fetal to adult stage. J Neurosci.

[16] Zecevic N, Bourgeois J-P, Rakic P (1989). Changes in synaptic density in motor cortex of rhesus monkey during fetal and postnatal life. Brain Res Dev Brain Res.

[17] Huttenlocher PR, de Courten C (1987). The development of synapses in striate cortex of man. Hum Neurobiol.

[18] Brown R, Colter N, Corsellis JA, Crow TJ, Frith CD, Jagoe R (1986). Postmortem evidence of structural brain changes in schizophrenia. Differences in brain weight, temporal horn area, and parahippocampal gyrus compared with affective disorder. Arch Gen Psychiatry.

[19] Pakkenberg B (1987). Post-mortem study of chronic schizophrenic brains. Br J Psychiatry.

[20] Andreasen N, Nasrallah HA, Dunn V, Olson SC, Grove WM, Ehrhardt JC (1986). Structural abnormalities in the frontal system in schizophrenia. A magnetic resonance imaging study. Arch Gen Psychiatry.

[21] DeMyer MK, Gilmor RL, Hendrie HC, DeMyer WE, Augustyn GT, Jackson RK (1988). Magnetic resonance brain images in schizophrenic and normal subjects: influence of diagnosis and education. Schizophr Bull.

[22] Rubin P, Karle A, Moller-Madsen S, Hertel C, Povlsen UJ, Noring U (1993). Computerised tomography in newly diagnosed schizophrenia and schizophreniform disorder. A controlled blind study. Br J Psychiatry.

[23] Zipursky RB, Lim KO, Sullivan EV, Brown BW, Pfefferbaum A (1992). Widespread cerebral gray matter volume deficits in schizophrenia. Arch Gen Psychiatry.

[24] Harvey I, Ron MA, Du Boulay G, Wicks D, Lewis SW, Murray RM (1993). Reduction of cortical volume in schizophrenia on magnetic resonance imaging. Psychol Med.

[25] Andreasen NC, Ehrhardt JC, Swayze VW, Alliger RJ, Yuh WT, Cohen G (1990). Magnetic resonance imaging of the brain in schizophrenia. The pathophysiologic significance of structural abnormalities. Arch Gen Psychiatry.

[26] Buchsbaum MS, Haier RJ (1987). Functional and anatomical brain imaging: impact on schizophrenia research. Schizophr Bull.

[27] Buchsbaum MS (1990). The frontal lobes, basal ganglia, and temporal lobes as sites for schizophrenia. Schizophr Bull.

[28] Buchsbaum MS, Haier RJ, Potkin SG, Nuechterlein K, Bracha HS, Katz M (1992). Frontostriatal disorder of cerebral metabolism in never-medicated schizophrenics. Arch Gen Psychiatry.

[29] Cleghorn JM, Garnett ES, Nahmias C, Firnau G, Brown GM, Kaplan R (1989). Increased frontal and reduced parietal glucose metabolism in acute untreated schizophrenia. Psychiatry Res.

[30] Jernigan TL, Zisook S, Heaton RK, Moranville JT, Hesselink JR, Braff DL (1991). Magnetic resonance imaging abnormalities in lenticular nuclei and cerebral cortex in schizophrenia. Arch Gen Psychiatry.

[31] Breier A, Buchanan RW, Elkashef A, Munson RC, Kirkpatrick B, Gellad F (1992). Brain morphology and schizophrenia. A magnetic resonance imaging study of limbic, prefrontal cortex, and caudate structures. Arch Gen Psychiatry.

[32] Brugger SP, Howes OD (2017). Heterogeneity and homogeneity of regional brain structure in schizophrenia: a meta-analysis. JAMA Psychiatry.

[33] Anderson SA, Classey JD, Conde F, Lund JS, Lewis DA (1995). Synchronous development of pyramidal neuron dendritic spines and parvalbumin-immunoreactive chandelier neuron axon terminals in layer III of monkey prefrontal cortex. Neuroscience.

[34] Petanjek Z, Judas M, Simic G, Rasin MR, Uylings HB, Rakic P (2011). Extraordinary neoteny of synaptic spines in the human prefrontal cortex. Proc Natl Acad Sci USA.

[35] Lyall AE, Shi F, Geng X, Woolson S, Li G, Wang L (2015). Dynamic development of regional cortical thickness and surface area in early childhood. Cereb Cortex.

[36] Tamnes CK, Herting MM, Goddings AL, Meuwese R, Blakemore SJ, Dahl RE (2017). Development of the cerebral cortex across adolescence: a multisample study of inter-related longitudinal changes in cortical volume, surface area, and thickness. J Neurosci.

[37] Mills KL, Goddings AL, Herting MM, Meuwese R, Blakemore SJ, Crone EA (2016). Structural brain development between childhood and adulthood: convergence across four longitudinal samples. Neuroimage.

[38] Norbom LB, Ferschmann L, Parker N, Agartz I, Andreassen OA, Paus T (2021). New insights into the dynamic development of the cerebral cortex in childhood and adolescence: integrating macro- and microstructural MRI findings. Prog Neurobiol.

[39] Bennett MR (2011). Schizophrenia: susceptibility genes, dendritic-spine pathology and gray matter loss. Prog Neurobiol.

[40] Paolicelli RC, Bolasco G, Pagani F, Maggi L, Scianni M, Panzanelli P (2011). Synaptic pruning by microglia is necessary for normal brain development. Science.

[41] Schafer DP, Lehrman EK, Kautzman AG, Koyama R, Mardinly AR, Yamasaki R (2012). Microglia sculpt postnatal neural circuits in an activity and complement-dependent manner. Neuron.

[42] Stevens B, Allen NJ, Vazquez LE, Howell GR, Christopherson KS, Nouri N (2007). The classical complement cascade mediates CNS synapse elimination. Cell.

[43] Yilmaz M, Yalcin E, Presumey J, Aw E, Ma M, Whelan CW (2021). Overexpression of schizophrenia susceptibility factor human complement C4A promotes excessive synaptic loss and behavioral changes in mice. Nat Neurosci.

[44] Druart M, Nosten-Bertrand M, Poll S, Crux S, Nebeling F, Delhaye C (2021). Elevated expression of complement C4 in the mouse prefrontal cortex causes schizophrenia-associated phenotypes. Mol Psychiatry.

[45] Chung WS, Allen NJ, Eroglu C (2015). Astrocytes control synapse formation, function, and elimination. Cold Spring Harb Perspect Biol.

[46] Chung WS, Clarke LE, Wang GX, Stafford BK, Sher A, Chakraborty C (2013). Astrocytes mediate synapse elimination through MEGF10 and MERTK pathways. Nature.

[47] Caroni P, Chowdhury A, Lahr M (2014). Synapse rearrangements upon learning: from divergent-sparse connectivity to dedicated sub-circuits. Trends Neurosci.

[48] Stein IS, Zito K (2019). Dendritic spine elimination: molecular mechanisms and implications. Neuroscientist.

[49] Uesaka N, Kano M (2018). Presynaptic mechanisms mediating retrograde semaphorin signals for climbing fiber synapse elimination during postnatal cerebellar development. Cerebellum.

[50] Trubetskoy V, Pardinas AF, Qi T, Panagiotaropoulou G, Awasthi S, Bigdeli TB (2022). Mapping genomic loci implicates genes and synaptic biology in schizophrenia. Nature.

[51] Purcell SM, Wray NR, Stone JL, Visscher PM, O’Donovan MC, International Schizophrenia Consortium (2009). Common polygenic variation contributes to risk of schizophrenia and bipolar disorder. Nature.

[52] Kim M, Haney JR, Zhang P, Hernandez LM, Wang LK, Perez-Cano L (2021). Brain gene co-expression networks link complement signaling with convergent synaptic pathology in schizophrenia. Nat Neurosci.

[53] Lam M, Chen CY, Li Z, Martin AR, Bryois J, Ma X (2019). Comparative genetic architectures of schizophrenia in East Asian and European populations. Nat Genet.

[54] Abdellaoui A, Dolan CV, Verweij KJH, Nivard MG (2022). Gene-environment correlations across geographic regions affect genome-wide association studies. Nat Genet.

[55] Cross-Disorder Group of the Psychiatric Genomics Consortium. (2019). Genomic relationships, novel loci, and pleiotropic mechanisms across eight psychiatric disorders. Cell.

[56] Schizophrenia Working Group of the Psychiatric Genomics Consortium. (2014). Biological insights from 108 schizophrenia-associated genetic loci. Nature.

[57] Kamiya A, Kubo K, Tomoda T, Takaki M, Youn R, Ozeki Y (2005). A schizophrenia-associated mutation of DISC1 perturbs cerebral cortex development. Nat Cell Biol.

[58] Jaaro-Peled H, Hayashi-Takagi A, Seshadri S, Kamiya A, Brandon NJ, Sawa A (2009). Neurodevelopmental mechanisms of schizophrenia: understanding disturbed postnatal brain maturation through neuregulin-1-ErbB4 and DISC1. Trends Neurosci.

[59] Stark KL, Xu B, Bagchi A, Lai WS, Liu H, Hsu R (2008). Altered brain microRNA biogenesis contributes to phenotypic deficits in a 22q11-deletion mouse model. Nat Genet.

[60] Mukai J, Dhilla A, Drew LJ, Stark KL, Cao L, MacDermott AB (2008). Palmitoylation-dependent neurodevelopmental deficits in a mouse model of 22q11 microdeletion. Nat Neurosci.

[61] Davenport EC, Szulc BR, Drew J, Taylor J, Morgan T, Higgs NF (2019). Autism and schizophrenia-associated CYFIP1 regulates the balance of synaptic excitation and inhibition. Cell Rep.

[62] De Rubeis S, Pasciuto E, Li KW, Fernandez E, Di Marino D, Buzzi A (2013). CYFIP1 coordinates mRNA translation and cytoskeleton remodeling to ensure proper dendritic spine formation. Neuron.

[63] Papaleo F, Yang F, Paterson C, Palumbo S, Carr GV, Wang Y (2016). Behavioral, neurophysiological, and synaptic impairment in a transgenic neuregulin1 (NRG1-IV) murine schizophrenia model. J Neurosci.

[64] Jia JM, Hu Z, Nordman J, Li Z (2014). The schizophrenia susceptibility gene dysbindin regulates dendritic spine dynamics. J Neurosci.

[65] Liu WS, Pesold C, Rodriguez MA, Carboni G, Auta J, Lacor P (2001). Down-regulation of dendritic spine and glutamic acid decarboxylase 67 expressions in the reelin haploinsufficient heterozygous reeler mouse. Proc Natl Acad Sci USA.

[66] Jiang DY, Wu Z, Forsyth CT, Hu Y, Yee SP, Chen G (2018). GABAergic deficits and schizophrenia-like behaviors in a mouse model carrying patient-derived neuroligin-2 R215H mutation. Mol Brain.

[67] Li Z, Chen J, Yu H, He L, Xu Y, Zhang D (2017). Genome-wide association analysis identifies 30 new susceptibility loci for schizophrenia. Nat Genet.

[68] Ikeda M, Takahashi A, Kamatani Y, Momozawa Y, Saito T, Kondo K (2019). Genome-wide association study detected novel susceptibility genes for schizophrenia and shared trans-populations/diseases genetic effect. Schizophr Bull.

[69] Khandaker GM, Zimbron J, Lewis G, Jones PB (2013). Prenatal maternal infection, neurodevelopment and adult schizophrenia: a systematic review of population-based studies. Psychol Med.

[70] Kępińska AP, Iyegbe CO, Vernon AC, Yolken R, Murray RM, Pollak TA (2020). Schizophrenia and influenza at the centenary of the 1918-1919 Spanish Influenza pandemic: mechanisms of psychosis risk. Front Psychiatry.

[71] Mirabella F, Desiato G, Mancinelli S, Fossati G, Rasile M, Morini R (2021). Prenatal interleukin 6 elevation increases glutamatergic synapse density and disrupts hippocampal connectivity in offspring. Immunity.

[72] Soumiya H, Fukumitsu H, Furukawa S (2011). Prenatal immune challenge compromises development of upper-layer but not deeper-layer neurons of the mouse cerebral cortex. J Neurosci Res.

[73] Meyer U (2013). Developmental neuroinflammation and schizophrenia. Prog Neuropsychopharmacol Biol Psychiatry.

[74] Silva-Gómez AB, Rojas DX, Juárez I, Flores G (2003). Decreased dendritic spine density on prefrontal cortical and hippocampal pyramidal neurons in postweaning social isolation rats. Brain Res.

[75] Comery TA, Shah R, Greenough WT (1995). Differential rearing alters spine density on medium-sized spiny neurons in the rat corpus striatum: evidence for association of morphological plasticity with early response gene expression. Neurobiol Learn Mem.

[76] Colyn L, Venzala E, Marco S, Perez-Otano I, Tordera RM (2019). Chronic social defeat stress induces sustained synaptic structural changes in the prefrontal cortex and amygdala. Behav Brain Res.

[77] Vyas A, Mitra R, Shankaranarayana Rao BS, Chattarji S (2002). Chronic stress induces contrasting patterns of dendritic remodeling in hippocampal and amygdaloid neurons. J Neurosci.

[78] Liston C, Miller MM, Goldwater DS, Radley JJ, Rocher AB, Hof PR (2006). Stress-induced alterations in prefrontal cortical dendritic morphology predict selective impairments in perceptual attentional set-shifting. J Neurosci.

[79] Radley JJ, Sisti HM, Hao J, Rocher AB, McCall T, Hof PR (2004). Chronic behavioral stress induces apical dendritic reorganization in pyramidal neurons of the medial prefrontal cortex. Neuroscience.

[80] Gilabert-Juan J, Bueno-Fernandez C, Castillo-Gomez E, Nacher J (2017). Reduced interneuronal dendritic arborization in CA1 but not in CA3 region of mice subjected to chronic mild stress. Brain Behav.

[81] Cieslik M, Gassowska-Dobrowolska M, Jesko H, Czapski GA, Wilkaniec A, Zawadzka A (2020). Maternal immune activation induces neuroinflammation and cortical synaptic deficits in the adolescent rat offspring. Int J Mol Sci.

[82] Li WY, Chang YC, Lee LJ, Lee LJ (2014). Prenatal infection affects the neuronal architecture and cognitive function in adult mice. Dev Neurosci.

[83] Kaul D, Smith CC, Stevens J, Frohlich AS, Binder EB, Mechawar N (2020). Severe childhood and adulthood stress associates with neocortical layer-specific reductions of mature spines in psychiatric disorders. Neurobiol Stress.

[84] Da Silva T, Guma E, Hafizi S, Koppel A, Rusjan P, Kennedy JL (2021). Genetically predicted brain C4A expression is associated with TSPO and hippocampal morphology. Biol Psychiatry.

[85] O’Connell KS, Sonderby IE, Frei O, van der Meer D, Athanasiu L, Smeland OB (2021). Association between complement component 4A expression, cognitive performance and brain imaging measures in UK Biobank. Psychol Med.

[86] Stanley JA, Pettegrew JW, Keshavan MS (2000). Magnetic resonance spectroscopy in schizophrenia: methodological issues and findings–part I. Biol Psychiatry.

[87] Prasad KM, Chowdari KV, D’Aiuto LA, Iyengar S, Stanley JA, Nimgaonkar VL (2018). Neuropil contraction in relation to Complement C4 gene copy numbers in independent cohorts of adolescent-onset and young adult-onset schizophrenia patients-a pilot study. Transl Psychiatry.

[88] Donohoe G, Holland J, Mothersill D, McCarthy-Jones S, Cosgrove D, Harold D (2018). Genetically predicted complement component 4A expression: effects on memory function and middle temporal lobe activation. Psychol Med.

[89] Osimo EF, Beck K, Reis Marques T, Howes OD (2019). Synaptic loss in schizophrenia: a meta-analysis and systematic review of synaptic protein and mRNA measures. Mol Psychiatry.

[90] Berdenis van Berlekom A, Muflihah CH, Snijders G, MacGillavry HD, Middeldorp J, Hol EM (2020). Synapse pathology in schizophrenia: a meta-analysis of postsynaptic elements in postmortem brain studies. Schizophr Bull.

[91] Roberts RC, Barksdale KA, Roche JK, Lahti AC (2015). Decreased synaptic and mitochondrial density in the postmortem anterior cingulate cortex in schizophrenia. Schizophr Res.

[92] Aganova EA, Uranova NA (1992). Morphometric analysis of synaptic contacts in the anterior limbic cortex in the endogenous psychoses. Neurosci Behav Physiol.

[93] Kung L, Conley R, Chute DJ, Smialek J, Roberts RC (1998). Synaptic changes in the striatum of schizophrenic cases: a controlled postmortem ultrastructural study. Synapse.

[94] Mabry SJ, McCollum LA, Farmer CB, Bloom ES, Roberts RC (2020). Evidence for altered excitatory and inhibitory tone in the post-mortem substantia nigra in schizophrenia. World J Biol Psychiatry.

[95] McCullumsmith RE, Hammond JH, Shan D, Meador-Woodruff JH (2014). Postmortem brain: an underutilized substrate for studying severe mental illness. Neuropsychopharmacology.

[96] Wang M, Zhang L, Gage FH (2020). Modeling neuropsychiatric disorders using human induced pluripotent stem cells. Protein Cell.

[97] Sellgren CM, Gracias J, Watmuff B, Biag JD, Thanos JM, Whittredge PB (2019). Increased synapse elimination by microglia in schizophrenia patient-derived models of synaptic pruning. Nat Neurosci.

[98] Sekar A, Bialas AR, de Rivera H, Davis A, Hammond TR, Kamitaki N (2016). Schizophrenia risk from complex variation of complement component 4. Nature.

[99] Bloomfield PS, Selvaraj S, Veronese M, Rizzo G, Bertoldo A, Owen DR (2016). Microglial activity in people at ultra high risk of psychosis and in schizophrenia: an [(11)C]PBR28 PET brain imaging study. Am J Psychiatry.

[100] Noh H, Shao Z, Coyle JT, Chung S (2017). Modeling schizophrenia pathogenesis using patient-derived induced pluripotent stem cells (iPSCs). Biochim Biophys Acta Mol Basis Dis.

[101] Shen X, Yeung HT, Lai KO (2019). Application of human-induced pluripotent stem cells (hiPSCs) to study synaptopathy of neurodevelopmental disorders. Dev Neurobiol.

[102] Kuo SS, Pogue-Geile MF (2019). Variation in fourteen brain structure volumes in schizophrenia: a comprehensive meta-analysis of 246 studies. Neurosci Biobehav Rev.

[103] Jalbrzikowski M, Hayes RA, Wood SJ, Nordholm D, Zhou JH, Enigma Clinical High Risk for Psychosis Working Group (2021). Association of structural magnetic resonance imaging measures with psychosis onset in individuals at clinical high risk for developing psychosis: an ENIGMA Working Group mega-analysis. JAMA Psychiatry.

[104] Cannon TD, Chung Y, He G, Sun D, Jacobson A, van Erp TG (2015). Progressive reduction in cortical thickness as psychosis develops: a multisite longitudinal neuroimaging study of youth at elevated clinical risk. Biol Psychiatry.

[105] Zhao Y, Zhang Q, Shah C, Li Q, Sweeney JA, Li F (2022). Cortical thickness abnormalities at different stages of the illness course in schizophrenia: a systematic review and meta-analysis. JAMA Psychiatry.

[106] Cropley VL, Klauser P, Lenroot RK, Bruggemann J, Sundram S, Bousman C (2017). Accelerated gray and white matter deterioration with age in schizophrenia. Am J Psychiatry.

[107] Vita A, De Peri L, Deste G, Sacchetti E (2012). Progressive loss of cortical gray matter in schizophrenia: a meta-analysis and meta-regression of longitudinal MRI studies. Transl Psychiatry.

[108] Vita A, De Peri L, Deste G, Barlati S, Sacchetti E (2015). The effect of antipsychotic treatment on cortical gray matter changes in schizophrenia: does the class matter? A meta-analysis and meta-regression of longitudinal magnetic resonance imaging studies. Biol Psychiatry.

[109] Ziermans TB, Schothorst PF, Schnack HG, Koolschijn PC, Kahn RS, van Engeland H (2012). Progressive structural brain changes during development of psychosis. Schizophr Bull.

[110] Padmanabhan JL, Tandon N, Haller CS, Mathew IT, Eack SM, Clementz BA (2015). Correlations between brain structure and symptom dimensions of psychosis in schizophrenia, schizoaffective, and psychotic bipolar I disorders. Schizophr Bull.

[111] Lui S, Deng W, Huang XQ, Jiang LJ, Ma XH, Chen HF (2009). Association of cerebral deficits with clinical symptoms in antipsychotic-naive first-episode schizophrenia: an optimized voxel-based morphometry and resting state functional connectivity study. Am J Psychiatry.

[112] Hulshoff Pol HE, Kahn RS (2008). What happens after the first episode? A review of progressive brain changes in chronically ill patients with schizophrenia. Schizophr Bull.

[113] Kassem MS, Lagopoulos J, Stait-Gardner T, Price WS, Chohan TW, Arnold JC (2013). Stress-induced grey matter loss determined by MRI is primarily due to loss of dendrites and their synapses. Mol Neurobiol.

[114] Keifer OP, Hurt RC, Gutman DA, Keilholz SD, Gourley SL, Ressler KJ (2015). Voxel-based morphometry predicts shifts in dendritic spine density and morphology with auditory fear conditioning. Nat Commun.

[115] Harrison PJ, Weinberger DR (2005). Schizophrenia genes, gene expression, and neuropathology: on the matter of their convergence. Mol Psychiatry.

[116] Selemon LD, Goldman-Rakic PS (1999). The reduced neuropil hypothesis: a circuit based model of schizophrenia. Biol Psychiatry.

[117] Garey LJ, Ong WY, Patel TS, Kanani M, Davis A, Mortimer AM (1998). Reduced dendritic spine density on cerebral cortical pyramidal neurons in schizophrenia. J Neurol Neurosurg Psychiatry.

[118] Glantz LA, Lewis DA (2000). Decreased dendritic spine density on prefrontal cortical pyramidal neurons in schizophrenia. Arch Gen Psychiatry.

[119] Glausier JR, Lewis DA (2013). Dendritic spine pathology in schizophrenia. Neuroscience.

[120] MacDonald ML, Alhassan J, Newman JT, Richard M, Gu H, Kelly RM (2017). Selective loss of smaller spines in schizophrenia. Am J Psychiatry.

[121] Davidsson P, Gottfries J, Bogdanovic N, Ekman R, Karlsson I, Gottfries C-G (1999). The synaptic-vesicle-specific proteins rab3a and synaptophysin are reduced in thalamus and related cortical brain regions in schizophrenic brains. Schizophr Res.

[122] Matosin N, Fernandez-Enright F, Lum JS, Engel M, Andrews JL, Gassen NC (2016). Molecular evidence of synaptic pathology in the CA1 region in schizophrenia. NPJ Schizophr.

[123] Eastwood SL, Harrison PJ (1995). Decreased synaptophysin in the medial temporal lobe in schizophrenia demonstrated using immunoautoradiography. Neuroscience.

[124] Glantz LA, Lewis DA (1997). Reduction of synaptophysin immunoreactivity in the prefrontal cortex of subjects with schizophrenia. Regional and diagnostic specificity. Arch Gen Psychiatry.

[125] May A, Gaser C (2006). Magnetic resonance-based morphometry: a window into structural plasticity of the brain. Curr Opin Neurol.

[126] Howes OD, Cummings C, Chapman GE, Shatalina E. Neuroimaging in schizophrenia: an overview of findings and their implications for synaptic changes. Neuropsychopharmacology. 2023;48:151–67. 10.1038/s41386-022-01426-x PMC9700830 36056106

[127] Weinberger DR, Radulescu E (2021). Structural magnetic resonance imaging all over again. JAMA Psychiatry.

[128] Finnema SJ, Nabulsi NB, Eid T, Detyniecki K, Lin SF, Chen MK (2016). Imaging synaptic density in the living human brain. Sci Transl Med.

[129] Nabulsi NB, Mercier J, Holden D, Carre S, Najafzadeh S, Vandergeten MC (2016). Synthesis and preclinical evaluation of 11C-UCB-J as a PET tracer for imaging the synaptic vesicle glycoprotein 2A in the brain. J Nucl Med.

[130] Lynch BA, Lambeng N, Nocka K, Kensel-Hammes P, Bajjalieh SM, Matagne A (2004). The synaptic vesicle protein SV2A is the binding site for the antiepileptic drug levetiracetam. Proc Natl Acad Sci USA.

[131] Bajjalieh SM, Frantz GD, Weimann JM, McConnell SK, Scheller RH (1994). Differential expression of synaptic vesicle protein 2 (SV2) isoforms. J Neurosci.

[132] Looney MR, Dohan FC, Davies KG, Seidenberg M, Hermann BP, Schweitzer JB (1999). Synaptophysin immunoreactivity in temporal lobe epilepsy-associated hippocampal sclerosis. Acta Neuropathol.

[133] Mutch SA, Kensel-Hammes P, Gadd JC, Fujimoto BS, Allen RW, Schiro PG (2011). Protein quantification at the single vesicle level reveals that a subset of synaptic vesicle proteins are trafficked with high precision. J Neurosci.

[134] Chen MK, Mecca AP, Naganawa M, Finnema SJ, Toyonaga T, Lin SF (2018). Assessing synaptic density in Alzheimer disease with synaptic vesicle glycoprotein 2A positron emission tomographic imaging. JAMA Neurol.

[135] Onwordi EC, Halff EF, Whitehurst T, Mansur A, Cotel M-C, Wells L (2020). Synaptic density marker SV2A is reduced in schizophrenia patients and unaffected by antipsychotics in rats. Nat Commun.

[136] Radhakrishnan R, Skosnik PD, Ranganathan M, Naganawa M, Toyonaga T, Finnema S (2021). In vivo evidence of lower synaptic vesicle density in schizophrenia. Mol Psychiatry.

[137] Mudge J, Miller NA, Khrebtukova I, Lindquist IE, May GD, Huntley JJ (2008). Genomic convergence analysis of schizophrenia: mRNA sequencing reveals altered synaptic vesicular transport in post-mortem cerebellum. PLoS One.

[138] Onwordi EC, Whitehurst T, Mansur A, Statton B, Berry A, Quinlan M (2021). The relationship between synaptic density marker SV2A, glutamate and N-acetyl aspartate levels in healthy volunteers and schizophrenia: a multimodal PET and magnetic resonance spectroscopy brain imaging study. Transl Psychiatry.

[139] Moyer CE, Shelton MA, Sweet RA (2015). Dendritic spine alterations in schizophrenia. Neurosci Lett.

[140] Rosso IM, Cannon TD, Huttunen T, Huttunen MO, Lonnqvist J, Gasperoni TL (2000). Obstetric risk factors for early-onset schizophrenia in a Finnish birth cohort. Am J Psychiatry.

[141] Howes OD, McCutcheon R (2017). Inflammation and the neural diathesis-stress hypothesis of schizophrenia: a reconceptualization. Transl Psychiatry.

[142] Giovanoli S, Engler H, Engler A, Richetto J, Voget M, Willi R (2013). Stress in puberty unmasks latent neuropathological consequences of prenatal immune activation in mice. Science.

[143] Tynan RJ, Naicker S, Hinwood M, Nalivaiko E, Buller KM, Pow DV (2010). Chronic stress alters the density and morphology of microglia in a subset of stress-responsive brain regions. Brain Behav Immun.

[144] Hinwood M, Morandini J, Day TA, Walker FR (2012). Evidence that microglia mediate the neurobiological effects of chronic psychological stress on the medial prefrontal cortex. Cereb Cortex.

[145] Calcia MA, Bonsall DR, Bloomfield PS, Selvaraj S, Barichello T, Howes OD (2016). Stress and neuroinflammation: a systematic review of the effects of stress on microglia and the implications for mental illness. Psychopharmacology (Berl).

[146] Wohleb ES, Fenn AM, Pacenta AM, Powell ND, Sheridan JF, Godbout JP (2012). Peripheral innate immune challenge exaggerated microglia activation, increased the number of inflammatory CNS macrophages, and prolonged social withdrawal in socially defeated mice. Psychoneuroendocrinology.

[147] Sohal VS, Zhang F, Yizhar O, Deisseroth K (2009). Parvalbumin neurons and gamma rhythms enhance cortical circuit performance. Nature.

[148] Howes OD, Shatalina E (2022). Integrating the neurodevelopmental and dopamine hypotheses of schizophrenia and the role of cortical excitation-inhibition balance. Biol Psychiatry.

[149] Townsend L, Pillinger T, Selvaggi P, Veronese M, Turkheimer F, Howes O. Brain glucose metabolism in schizophrenia: a systematic review and meta-analysis of (18)FDG-PET studies in schizophrenia. Psychol Med. 2022:1–18. 10.1017/S003329172200174X PMC10476075 35730361

[150] Friston K, Brown HR, Siemerkus J, Stephan KE (2016). The dysconnection hypothesis (2016). Schizophr Res.

[151] Fornito A, Harrison BJ, Goodby E, Dean A, Ooi C, Nathan PJ (2013). Functional dysconnectivity of corticostriatal circuitry as a risk phenotype for psychosis. JAMA Psychiatry.

[152] Cui LB, Liu K, Li C, Wang LX, Guo F, Tian P (2016). Putamen-related regional and network functional deficits in first-episode schizophrenia with auditory verbal hallucinations. Schizophr Res.

[153] White TP, Wigton R, Joyce DW, Collier T, Fornito A, Shergill SS (2016). Dysfunctional striatal systems in treatment-resistant schizophrenia. Neuropsychopharmacology.

[154] Shukla DK, Chiappelli JJ, Sampath H, Kochunov P, Hare SM, Wisner K (2019). Aberrant frontostriatal connectivity in negative symptoms of schizophrenia. Schizophr Bull.

[155] O’Neill A, Mechelli A, Bhattacharyya S (2019). Dysconnectivity of large-scale functional networks in early psychosis: a meta-analysis. Schizophr Bull.

[156] Lord LD, Allen P, Expert P, Howes O, Lambiotte R, McGuire P (2011). Characterization of the anterior cingulate’s role in the at-risk mental state using graph theory. Neuroimage.

[157] Zhang K, Huang D, Shah NJ (2018). Comparison of resting-state brain activation detected by BOLD, blood volume and blood flow. Front Hum Neurosci.

[158] McCutcheon RA, Krystal JH, Howes OD (2020). Dopamine and glutamate in schizophrenia: biology, symptoms and treatment. World Psychiatry.

[159] Quiroz C, Orru M, Rea W, Ciudad-Roberts A, Yepes G, Britt JP (2016). Local control of extracellular dopamine levels in the medial nucleus accumbens by a glutamatergic projection from the infralimbic cortex. J Neurosci.

[160] Kim IH, Rossi MA, Aryal DK, Racz B, Kim N, Uezu A (2015). Spine pruning drives antipsychotic-sensitive locomotion via circuit control of striatal dopamine. Nat Neurosci.

[161] Kokkinou M, Irvine EE, Bonsall DR, Natesan S, Wells LA, Smith M (2021). Reproducing the dopamine pathophysiology of schizophrenia and approaches to ameliorate it: a translational imaging study with ketamine. Mol Psychiatry.

[162] D’Ambrosio E, Jauhar S, Kim S, Veronese M, Rogdaki M, Pepper F (2021). The relationship between grey matter volume and striatal dopamine function in psychosis: a multimodal (18)F-DOPA PET and voxel-based morphometry study. Mol Psychiatry.

[163] Howes OD, Hird EJ, Adams RA, Corlett PR, McGuire P (2020). Aberrant salience, information processing, and dopaminergic signaling in people at clinical high risk for psychosis. Biol Psychiatry.

[164] Cahn W, Rais M, Stigter FP, van Haren NE, Caspers E, Hulshoff Pol HE (2009). Psychosis and brain volume changes during the first five years of schizophrenia. Eur Neuropsychopharmacol.

[165] Howes OD, Whitehurst T, Shatalina E, Townsend L, Onwordi EC, Mak TLA (2021). The clinical significance of duration of untreated psychosis: an umbrella review and random-effects meta-analysis. World Psychiatry.

[166] van Kesteren CF, Gremmels H, de Witte LD, Hol EM, Van Gool AR, Falkai PG (2017). Immune involvement in the pathogenesis of schizophrenia: a meta-analysis on postmortem brain studies. Transl Psychiatry.

[167] Marques TR, Ashok AH, Pillinger T, Veronese M, Turkheimer FE, Dazzan P, et al. Neuroinflammation in schizophrenia: meta-analysis of in vivo microglial imaging studies. Psychol Med. 2019;49:2186–96. 10.1017/S0033291718003057 PMC6366560 30355368

[168] Plaven-Sigray P, Matheson GJ, Collste K, Ashok AH, Coughlin JM, Howes OD (2018). Positron emission tomography studies of the glial cell marker translocator protein in patients with psychosis: a meta-analysis using individual participant data. Biol Psychiatry.

[169] Kopschina Feltes P, de Vries EF, Juarez-Orozco LE, Kurtys E, Dierckx RA, Moriguchi-Jeckel CM (2019). Repeated social defeat induces transient glial activation and brain hypometabolism: a positron emission tomography imaging study. J Cereb Blood Flow Metab.

[170] McCluskey SP, Plisson C, Rabiner EA, Howes O (2020). Advances in CNS PET: the state-of-the-art for new imaging targets for pathophysiology and drug development. Eur J Nucl Med Mol Imaging.

[171] Gracias J, Orhan F, Horbeck E, Holmen-Larsson J, Khanlarkani N, Malwade S (2022). Cerebrospinal fluid concentration of complement component 4A is increased in first episode schizophrenia. Nat Commun.

[172] Wolfers T, Doan NT, Kaufmann T, Alnaes D, Moberget T, Agartz I (2018). Mapping the heterogeneous phenotype of schizophrenia and bipolar disorder using normative models. JAMA Psychiatry.

[173] Kane JM, Agid O, Baldwin ML, Howes O, Lindenmayer JP, Marder S (2019). Clinical guidance on the identification and management of treatment-resistant schizophrenia. J Clin Psychiatry.

[174] Jauhar S, Veronese M, Nour MM, Rogdaki M, Hathway P, Turkheimer FE (2019). Determinants of treatment response in first-episode psychosis: an (18)F-DOPA PET study. Mol Psychiatry.

[175] Veronese M, Santangelo B, Jauhar S, D’Ambrosio E, Demjaha A, Salimbeni H (2021). A potential biomarker for treatment stratification in psychosis: evaluation of an [(18)F] FDOPA PET imaging approach. Neuropsychopharmacology.

[176] Jaaskelainen E, Juola P, Hirvonen N, McGrath JJ, Saha S, Isohanni M (2013). A systematic review and meta-analysis of recovery in schizophrenia. Schizophr Bull.

[177] Brennand KJ, Simone A, Jou J, Gelboin-Burkhart C, Tran N, Sangar S (2011). Modelling schizophrenia using human induced pluripotent stem cells. Nature.

[178] Wen Z, Nguyen HN, Guo Z, Lalli MA, Wang X, Su Y (2014). Synaptic dysregulation in a human iPS cell model of mental disorders. Nature.

[179] Shao Z, Noh H, Bin Kim W, Ni P, Nguyen C, Cote SE (2019). Dysregulated protocadherin-pathway activity as an intrinsic defect in induced pluripotent stem cell-derived cortical interneurons from subjects with schizophrenia. Nat Neurosci.

[180] Kathuria A, Lopez-Lengowski K, Watmuff B, McPhie D, Cohen BM, Karmacharya R (2019). Synaptic deficits in iPSC-derived cortical interneurons in schizophrenia are mediated by NLGN2 and rescued by N-acetylcysteine. Transl Psychiatry.

[181] Grunwald LM, Stock R, Haag K, Buckenmaier S, Eberle MC, Wildgruber D (2019). Comparative characterization of human induced pluripotent stem cells (hiPSC) derived from patients with schizophrenia and autism. Transl Psychiatry.

